# No change in plasma tau and serum neurofilament light concentrations in adolescent athletes following sport-related concussion

**DOI:** 10.1371/journal.pone.0206466

**Published:** 2018-10-29

**Authors:** Colin Wallace, Henrik Zetterberg, Kaj Blennow, Paul van Donkelaar

**Affiliations:** 1 School of Health and Exercise Sciences, University of British Columbia, Kelowna, British Columbia, Canada; 2 Department of Pathology and Laboratory Medicine, University of British Columbia, Vancouver, British Columbia, Canada; 3 Clinical Neurochemistry Laboratory, Sahlgrenska University Hospital, Mölndal, Sweden; 4 Department of Psychiatry and Neurochemistry, Institute of Neuroscience and Physiology, Sahlgrenska Academy at University of Gothenburg, Sahlgrenska University Hospital, Mölndal, Sweden; 5 Department of Molecular Neuroscience, UCL Institute of Neurology, London, United Kingdom; 6 UK Dementia Research Institute at UCL, London, United Kingdom; Case Western Reserve University, UNITED STATES

## Abstract

Sport-related concussion (SRC), a mild form of traumatic brain injury (TBI), is a common injury in contact sports. Health care professionals rely on subjective criteria (e.g., symptoms), as there is no objective marker for identification of athletes with SRC. Blood-based biomarkers have shown promise as diagnostic and prognostic tools following TBI and SRC. In the present study, we examined plasma tau and serum NF-L, two biomarkers for neuronal/axonal injury, concentrations at preseason and following SRC in contact sport athletes (*n* = 11) using ultrasensitive single molecule array (Simoa) assays. Preseason baseline samples were collected, and post-concussion samples were obtained at 6- and 14-days following injury. We found no difference between baseline, 6-day and 14-day post-concussion concentrations of tau (*p* = 0.14) or NF-L (*p* = 0.53). Further, no difference was found between preseason baseline and all post-SRC samples for tau (*p* = 0.22) or NF-L (*p* = 0.98). The total number of symptoms reported on the Standardized Assessment of Concussion– 3^rd^ Edition (SCAT3) and associated symptom severity scores increased from preseason to 6-days post-SRC but returned to baseline values at 14-days (*p* = 0.02 and *p* = 0.003, respectively). These results suggest that the severity of neuronal injury in this cohort of contact sport athletes with clinical uncomplicated SRC was too low to be detected by tau and NF-L measurements in blood samples obtained at 6- and 14-days post-injury.

## Introduction

Traumatic brain injury (TBI) is a growing public health concern: upwards of 3.8 million TBIs, including sport-related concussions (SRC), occur each year in the United States alone.[1] The incidence of SRC is much higher in contact sport athletes (e.g., American football, rugby, ice hockey, etc.) than in non-contact sport athletes (e.g., track and field).[2–5] SRC symptoms are highly variable and largely subjective, and affect somatic, cognitive, and emotional systems following injury.[6] Therefore, SRC is challenging to detect and diagnose, as there remains no objective diagnostic tool for either the acute or chronic stage.[7] SRC diagnosis is based on clinical criteria, including athlete-reported symptoms, cognitive function screening, and balance testing.[6] Improvements in symptom severity, balance deficits, and cognitive dysfunction guide return-to-play (RTP) progression with the majority of athletes who suffer a SRC being cleared to RTP within 2 weeks of injury.[6] However, executive dysfunction is shown to persist no less than 2 months post-injury,[8] suggesting clinical recovery and RTP occurs prior to full functional recovery. An objective diagnostic test in the acute phase following SRC used in conjunction with clinical symptom resolution would better ensure athletes RTP at the appropriate time.

Many sports require rapid decision making through complex mental operations. As such, separation of clinical and physiological recovery may result in a greater risk of injury due to cognitive impairments. Indeed, SRC increases the risk of lower body orthopaedic injury in collegiate athletes up to 1-year post-concussion compared to before the SRC occurred, and when compared to a group of nonconcussed athletes.[9] Reaction time is also increased following SRC in tasks engaging executive function networks,[8,10] offering a possible explanation for increased rates of lower body orthopaedic injury. Taken together, this suggests that functional deficits reflect an underlying pathology that cannot be accurately defined by current subjective criteria.

In addition to the short-term effects of a concussive injury, repetitive concussive and subconcussive impacts sustained over many years are proposed mechanisms for the development of certain neurodegenerative conditions, specifically chronic traumatic encephalopathy (CTE).[11] CTE is a progressive neurodegenerative condition with clinical symptoms typically presenting several years after removal from head impact exposure,[12] and diagnosis is possible only at post-mortem evaluation.[11] Recently, an *in vivo* neural imaging method of detecting CTE through the presence of unique positron emission tomography (PET) [F18]FDDNP binding patterns was proposed through a case study involving 1 retired National Football League player;[13] however, more research is required to establish the clinical application of this modality. No *in vivo* objective markers are currently available to quantify the extent of neuronal or glial damage in the brain due to head trauma, nor are there tools for determining physiological recovery from these impacts.[7]

Fluid-based biomarkers have been investigated as potential indicators of SRC, with both tau and neurofilament light (NF-L) hypothesized to be optimal markers of axonal damage.[7] Tau is a CNS-enriched microtubule-associated protein predominantly expressed in thin, unmyelinated axons and is involved in the formation and stability of microtubules.[14] Levels of total tau in cerebrospinal fluid (CSF) increase dramatically following acute TBI, with the degree of increase predicting long-term clinical outcome.[15] Plasma levels of total tau (T-tau) have been shown to be elevated up to 144-hours following concussion in professional ice hockey players compared to preseason baseline samples in uninjured controls, with the highest concentration measured 1-hour post-injury.[16] Similarly, Neselius *et al*. (2013) showed increased plasma T-tau in amateur boxers after a bout compared to controls.[17] Interestingly, Gill *et al*. (2017) found lower levels of plasma tau in concussed athletes at 24 and 72 hours post-injury when compared to nonconcussed athletes sampled at similar time points, although both concussed and nonconcussed athletes had higher levels of plasma tau compared to healthy controls with no history of head trauma.[18] This suggests a possible effect of exercise on circulating plasma tau concentration. Indeed, Shahim *et al*. (2018) found elevated levels of plasma tau 1-hour following exercise; however, concentration returned to baseline levels at 12-hours following exertion. [19] This should be considered when examining samples from early time points in acute concussed athletes.

NF-L is a CNS-enriched protein highly expressed in myelinated, long-caliber subcortical axons and contributes to the strength of the axon.[7] Following concussion, serum NF-L is increased in professional ice hockey players compared to uninjured controls and follows an upward trajectory with the highest concentrations seen at 12- and 144-hours post-injury.[20] After severe TBI, serum NF-L concentration peaks at 10–12 days.[21] Serum NF-L is also increased at 7–10 days in amateur boxers with no clinically diagnosed concussion following a match[20] and at the conclusion of an American football season compared to preseason values,[22] suggesting that repetitive, subconcussive impacts also cause damage to axonal structures. Unlike plasma tau, serum NF-L concentration is not affected by physical exertion. [19]

An objective method of concussion detection must be developed that is sensitive, measureable, reliable, repeatable, quick, and cost-effective.[7] A significant, sustained increase in the biomarker is required to truly distinguish between concussed and nonconcussed individuals. In the present study, we examined the dynamics of tau and NF-L in response to SRC in a cohort of junior and collegiate-aged American football and hockey athletes with the expressed interest in extending the temporal profile of these proteins post-concussion. Increased concentrations of these proteins 2 weeks post-concussion may reflect continuous release from the brain parenchyma, indicating prolonged axonal breakdown.

## Methods

### Study population and blood sample processing

This prospective cohort study enrolled 142 male American football (*n* = 94) and ice hockey (*n* = 48) players in Kelowna, British Columbia, Canada. Blood samples were collected by standard venipuncture through a vein in the antecubital fossa into one K_2_EDTA tube for plasma and one serum separator tube. Plasma samples were kept on ice until centrifugation, which occurred within 60 minutes of collection. Serum samples collected in the laboratory at the University of British Columbia were kept at room temperature for 30 minutes to allow for coagulation. Serum samples collected on site at the athletic training facility for each team were transported to the University of British Columbia on ice, removed, and kept at room temperature for 60 minutes to allow for coagulation. All samples were centrifuged at 1200*g* for 15 minutes, aliquoted, and stored at -80°C until the end of the study period. Once the study had ended, all samples were shipped on dry ice to Sahlgrenska University Hospital at the University of Gothenburg, Sweden for analysis. This study was approved by the Clinical Research Ethics Board at the University of British Columbia, Canada and was conducted in accordance to principles expressed in the Declaration of Helsinki. Written informed consent was obtained from all participants aged 18 or over, and written informed assent was obtained from the guardians of athletes under the age of 18.

### Blood sampling and quantification procedures

We collected preseason blood samples from all 142 male contact sport athletes. 13 (mean age 18.5 years ± 1.7) were subsequently diagnosed with a concussion, which was confirmed by team medical personnel using the Standardized Concussion Assessment Tool– 3^rd^ Edition (SCAT3) and consented to serial blood draws at 6- and 14-days post-injury. Athletes did not report a secondary injury at the time of concussion, and no loss of consciousness (LOC) was reported. All athletes returned to play within 3 weeks of injury. Eight players had previously suffered a concussion during their athletic career with an average of 1.1 ± 1.1 concussions for the group. We chose the 6- and 14-day time points to extend the temporal profile of these proteins and build on their previously reported kinetics.[16,20]

Quantification of plasma tau and serum NF-L occurred on the single molecule array (Simoa) HD-1 analyzer (Quanterix, Lexington, MA, USA), as previously described.[16,23] The Simoa HD-1 analyzer platform isolates individual capture beads in arrays of femtoliter-sized reaction wells, resulting in assay sensitivity 1000x greater than conventional enzyme linked immunosorbent assay (ELISA) techniques.[24,25] Lower limit of quantification (LLOQ) values 1.22 pg/mL and 0.58 pg/mL for tau and NF-L, respectively, using standard 4-fold sample dilutions. Samples were run in duplicate over two lots with samples from the same athlete run on the same plate by board-certified technicians blinded to clinical information. The coefficients of variation for all samples reported were < 20%.

### Statistical analysis methods

Statistical analyses were conducted with Statistical Package for the Social Sciences (SPSS) version 23 (IBM Corporation, Armonk, NY) and figures were developed using GraphPad Prism version 7.0 (GraphPad Software, San Diego, CA). We compared tau and NF-L concentrations across 3 time points (preseason, 6-days post-concussion, 14-days post-concussion) using separate repeated measures analyses of variance (rmANOVA) for each protein. A Mann-Whitney *U* test was run to determine if there were differences in tau concentration from preseason to post-concussion as the data were not normally distributed. Due to the normality of the NF-L data, an independent-samples t-test was run to determine if there were differences in concentration from preseason to post-concussion. A Friedman test was run to determine if there were differences in the total number of symptoms reported on the SCAT3 and symptom severity score as the data were not normally distributed. Pairwise comparisons were performed with a Bonferroni correction for multiple comparisons. A rmANOVA was used to determine whether there were statistically significant differences in the total number of errors reported on the Balance Error Scoring System (BESS) and Standardized Assessment of Concussion score.

## Results

### Participants and blood samples

**Fig 1:**
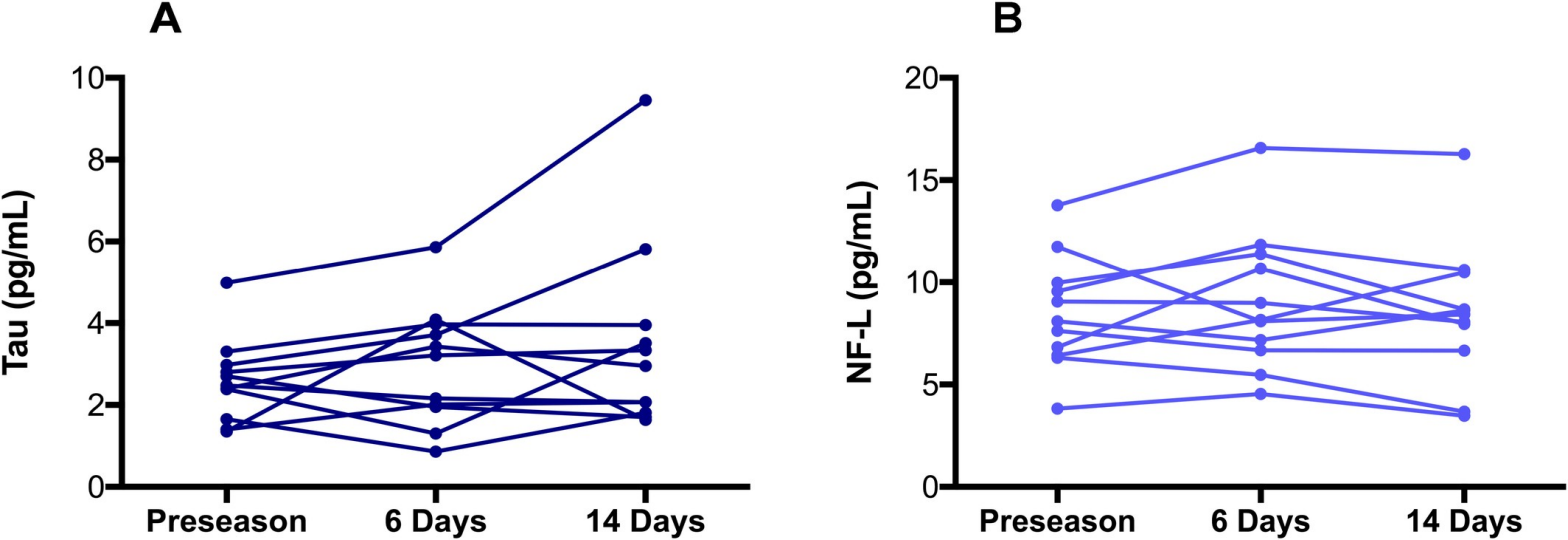
Tau and NF-L concentrations do not change following uncomplicated SRC. (A) Tau concentrations at baseline, and at 6- and 14-days following uncomplicated SRC. (B) NF-L concentrations at baseline, and at 6- and 14-days following uncomplicated SRC.

**Fig 2:**
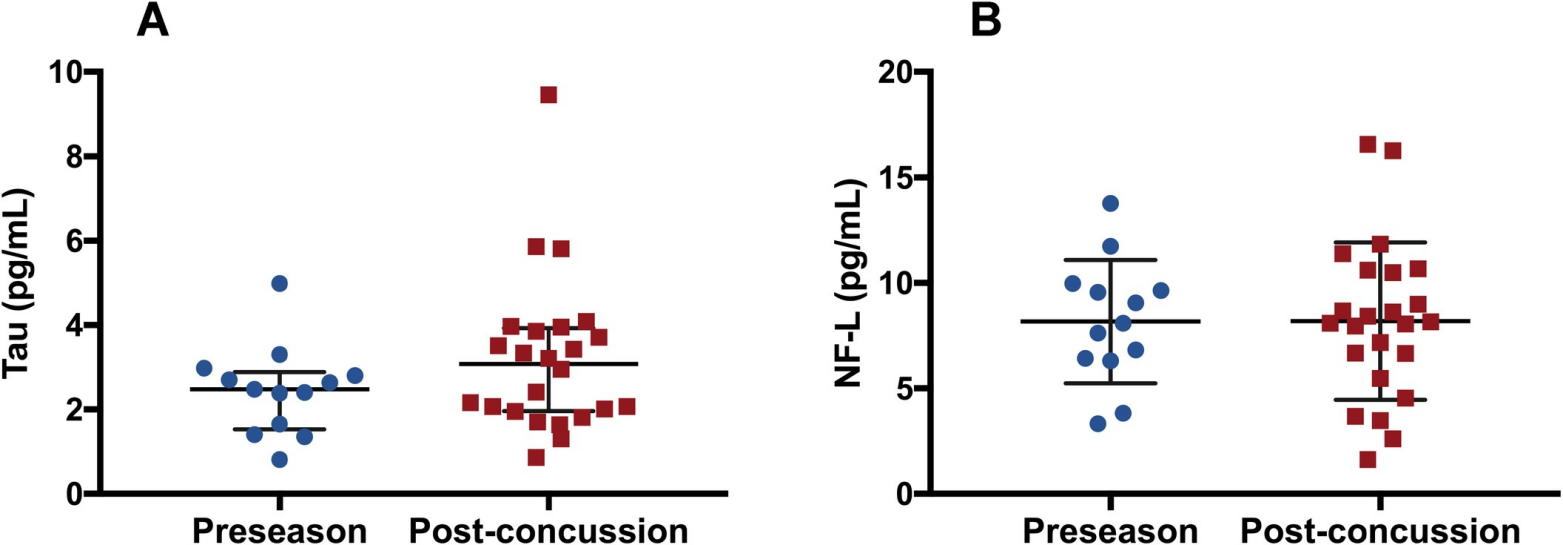
Tau and NF-L concentrations at preseason and following uncomplicated SRC (all time points). (A) Tau concentrations were unchanged in post-concussion samples (all times) compared to preseason samples. (B) There was no difference between preseason and post-concussion (all times) NF-L concentrations.

Table 1 provides biomarker information on the subjects in this study. Two participants were unable to provide 6-day follow-up blood samples due to travel, and so protein concentrations were removed from repeated measures analyses. Although there were no significant changes in biomarker concentrations following concussion, mean tau concentration for the remaining 11 athletes was 2.56 ± 1.02, 2.96 ± 1.46 pg/mL and 3.48 ± 2.34 pg/mL at baseline, 6-days post-injury and 14-days post-injury (Fig 1A), respectively. Mean NF-L concentration was 8.47 ± 2.77 pg/mL at baseline, 9.05 ± 3.42 pg/mL and 8.45 ± 3.48 pg/mL at 6- and 14-days (Fig 1B), respectively. rmANOVA analysis revealed no significant differences across time for both tau (*F*(2, 20) = 2.184, *p* = 0.14) and NF-L (*F*(2, 20) = 0.654, *p* = 0.53) (Fig 2A and 2B).

**Table 1:** Biomarker concentrations and SCAT3 metrics before and after sport-related concussion.

Characteristic	Time			*p*-value
	Preseason	6 Days	14 Days	
Tau (pg/mL)	2.56 ± 1.02	2.96 ± 1.46	3.48 ± 2.34	0.14
NF-L (pg/mL)	8.47 ± 2.77	9.05 ± 3.42	8.45 ± 3.48	0.53
Number of symptoms reported	1 (0–2)	16 (3–19)	0 (0–2)	**0.02**
Symptom severity	1 (0–5)	16 (6–60)	0 (0–4)	**0.003**
BESS total errors	3.71 ± 2.29	4.71 ± 3.30	2.43 ± 2.64	0.18
SAC total	26.71 ± 1.89	25.00 ± 2.08	25.86 ± 2.61	0.31

BESS, balance error scoring system; NF-L, neurofilament light; SAC, standardized assessment of concussion; SRC, sport-related concussion

All data are presented as mean ± standard deviation, except SCAT3 number of symptoms and symptom severity, which are presented as median (range).

Median tau preseason (2.48 pg/mL) and all post-concussion (3.08 pg/mL) concentrations were not statistically significantly different, *U* = 195, z = 1.241, *p* = 0.22 (Fig 2A). There was no difference between preseason (mean ± SD, 8.17 ± 2.93 pg/mL) and all post-concussion (mean ± SD, 8.20 ± 3.74 pg/mL) NF-L concentrations, *t*(35) = -0.024, *p* = 0.98 (Fig 2B).

### SCAT3 results

**Fig 3:**
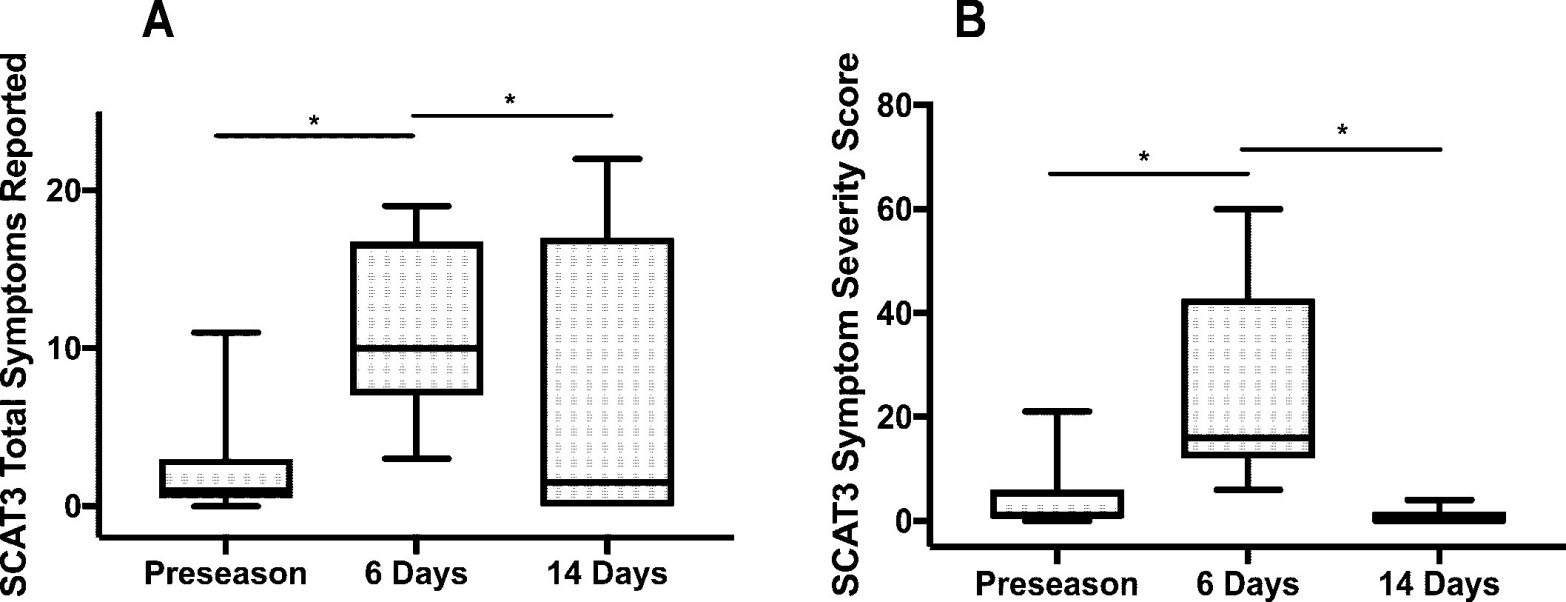
Total number of symptoms and symptom severity scores assessed by SCAT3 at preseason and following uncomplicated SRC. (A) The total number of symptoms reported on the SCAT3 increased from preseason to 6-days following uncomplicated SRC and returned to baseline at 14-days. Maximum possible number of symptoms is 22. (B) Symptom severity scores increased at 6-days following uncomplicated SRC compared to baseline and returned to baseline at 14-days following injury. Maximum possible symptom severity score is 132.

**Fig 4:**
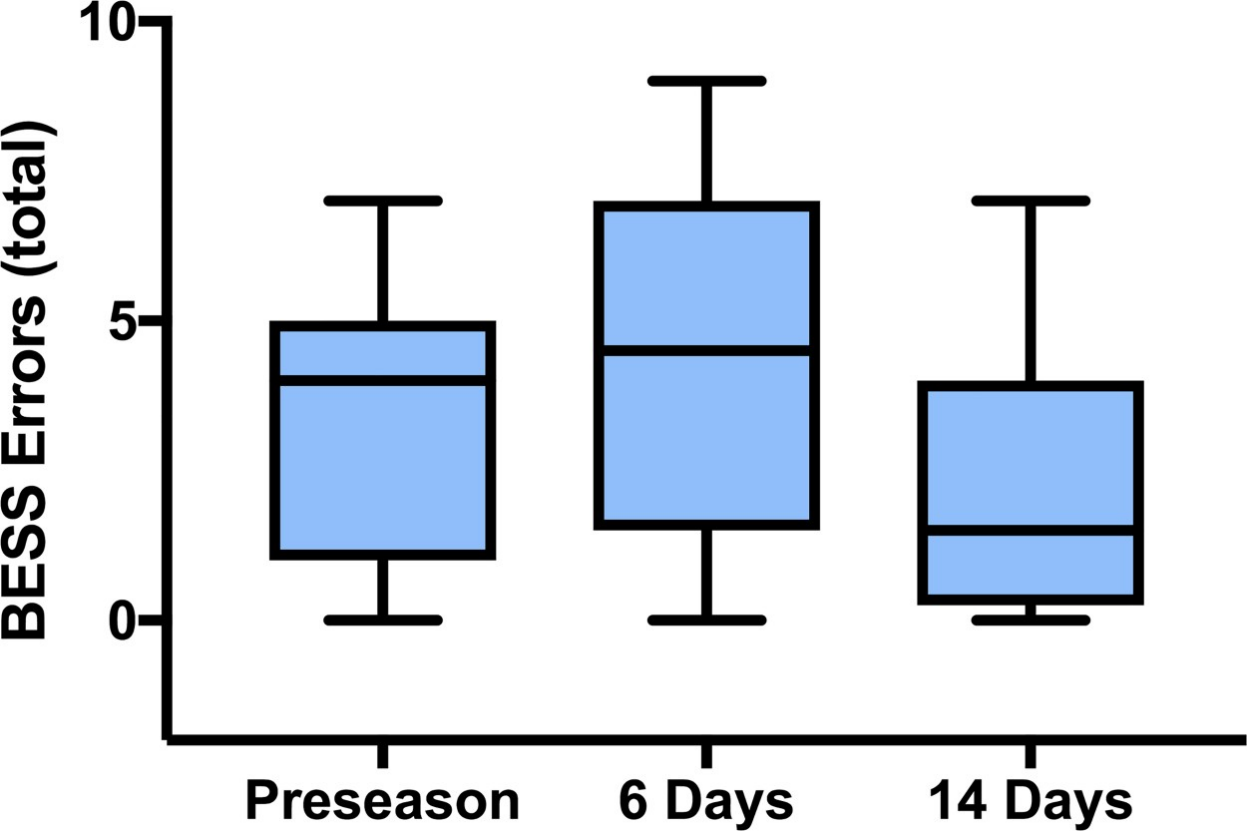
Total number of errors on the balance error scoring system (BESS) at preseason, and 6- and 14-days following uncomplicated SRC. The total number of errors assessed by the BESS was unchanged following uncomplicated SRC compared to preseason values. The BESS is calculated by adding one point per error during 3 separate 20-second tests.

**Fig 5:**
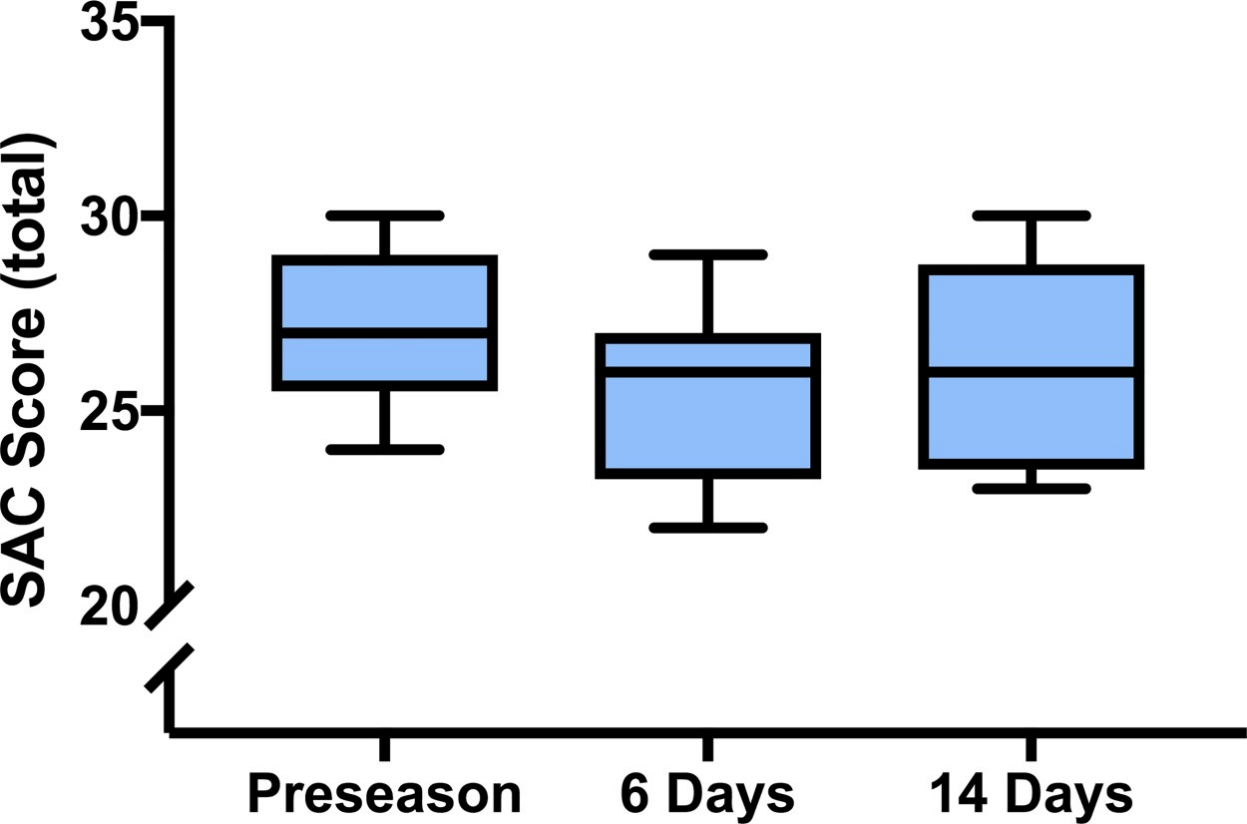
Total number of correct answers on the standardized assessment of concussion (SAC) at preseason, and 6- and 14-days following uncomplicated SRC. Total number of correct answers, out of 31, was unchanged following uncomplicated SRC compared to preseason values.

Sport-related concussion elicited a statistically significant difference in the total number of symptoms reported, χ^2^(2) = 7.895, *p* = 0.02, and symptom severity score, χ^2^(2) = 11.385, *p* < 0.01 (Fig 3A and 3B). Post-hoc analysis revealed a statistically significant increase in the total number of symptoms reported from preseason (*Mdn* = 1) to 6-days post-concussion (*Mdn* = 16) (*p* = 0.05), and a return to baseline levels at 14-days following injury (*Mdn* = 0; *p* = 0.05). Post-hoc analysis revealed a statistically significant increase in symptom severity score from preseason (*Mdn* = 1) to 6-days post-concussion (*Mdn* = 16) (*p* = 0.02). Symptom severity score values returned to baseline levels at 14-days post-concussion (*Mdn* = 0; *p* = 0.01). There was no statistically significant difference between total number of errors on the BESS (*p* = 0.18) and SAC total (*p* = 0.31) (Figs 4 and 5).

## Discussion

To our knowledge, our study is the first to examine baseline and post-SRC samples from the same individuals. All documented SRC included in the present study were considered uncomplicated, as all athletes began a graduated RTP protocol within 10-days of injury upon resolution of symptoms and returned to play within 17-days following injury. Uncomplicated SRC resulted in no significant increase in plasma tau and serum NF-L at 6- and 14-days post-concussion relative to their baseline concentrations (Fig 1). Further, no significant differences were found between preseason and all post-concussion concentrations for tau (Fig 2A) or NF-L (Fig 2B).

Sport-related concussion is caused by an external force to the body transmitted to the head,[6] after which a variety of cognitive (e.g., feeling like in a fog), somatic (e.g., headache) and emotional (e.g., rapid changes in mood) symptoms arise. The degree of increase in plasma tau concentration at 1-hour following SRC is correlated with the length of time to symptom resolution[16] and the degree of symptom severity.[19] Further, Shahim *et al*. (2014) found elevated plasma tau in concussed professional hockey players with post-concussion symptoms (PCS) lasting longer than 6-days compared to concussed players with PCS lasting less than 6-days,[16] and Gill *et al*. (2017) found elevated plasma tau in concussed athletes experiencing longer RTP timelines than concussed athletes with a relatively short RTP.[18] Serum NF-L is elevated in athletes with PCS lasting longer than 6-days; however, serum NF-L concentration mirrors that of uninjured controls in athletes who report full resolution of symptoms within 6-days of SRC.[20] Therefore, a detectable increase in both proteins may reflect more widespread axonal trauma. As such, it has been hypothesized that quantitation of these biomarkers in blood can be used as an objective clinical tool to detect complicated SRC (i.e., SRC with symptoms lasting longer than 6-days). The data in the present study suggest that plasma tau and serum NF-L may not be sensitive enough to detect uncomplicated SRC at 6- and 14-days post-injury in adolescent athletes that experience a rapid RTP; this could be attributed to a lower severity of axonal damage than in previous studies.[16–18]

One athlete in the present study experienced an elevation in both plasma tau and serum NF-L at 6-days compared to baseline, and again at 14-days compared to 6-days. At 6-days post-concussion this subject reported 19 total symptoms on the SCAT3 with a symptom severity score of 60. This was increased compared to median group values of number of symptoms (i.e., 10) and symptom severity (i.e., 16), indicating the possibility of a more serious injury; however, this athlete had a full RTP in 12 days, reported no symptoms at 14-days post-injury, and BESS errors (i.e., 7) and SAC total (i.e., 25) at 14-days post-injury mirrored his preseason values. No significant differences were seen between this player and the remaining 10 athletes concerning age and concussion history (i.e., 1 prior concussion).

Cognitive and balance assessments on the SCAT3 were performed immediately before each blood draw. The SAC is a clinically validated tool for the assessment of SRC;[26] however, players reevaluated with the SAC at 48 hours post-injury and beyond have reported similar scores to baseline values.[27,28] Athletes in the present study completed the SAC well outside this time point following injury and no differences in score were found, highlighting the importance of evaluating athletes for SRC as close to the time of injury as possible. The BESS has not shown consistent reliability across the literature[29] and a learning effect has been reported in athletes.[30] Healthcare practitioners should be cautious when performing this assessment post-SRC, especially if administered on several occasions, as practice effects could mask any abnormalities that may be present.

Tau and NF-L are key structural components of grey-matter and white-matter axons, respectively, and their quantification in blood following a suspected SRC may help health care professionals accurately identify and grade neuronal injury in a concussed athlete. Indeed, several researchers have shown increases in both plasma tau and serum NF-L following SRC compared to baseline control.[16–18,20] With respect to tau, these increases are more pronounced in the initial hours following injury,[16,18] whereas NF-L appears to have a slower release into circulating blood.[20] Further, serum NF-L levels correlate with both cerebrospinal fluid (CSF) levels following severe TBI,[21] and magnetic resonance diffusion tensor imaging abnormalities 12 months following diffuse axonal injury (DAI).[31] Similar trends have been found for other analytes, including glial fibrillary acidic protein,[32] enzyme-cleaved fragments of tau,[33] and calpain-derived αII-spectrin.[34] As no fluid-based biomarker test for SRC has been approved for clinical use, diagnosis remains focused on cognitive function, balance testing, and symptoms. If a SRC is suspected, and an athlete reports any symptom associated with a SRC, the athlete should be removed from play immediately; however, athletes may not report symptoms immediately following injury[35], and so it is imperative that research in this area continues so as to identify blood-based biomarkers that can detect SRC across the entire spectrum of severity.

In conclusion, our study suggests that uncomplicated SRC is not associated with increases in plasma tau or serum NF-L concentrations at 6- and 14-days following injury. The diagnosis of SRC is difficult, as it is primarily based on athlete-reported symptoms and these can vary greatly in time to onset, duration and severity, making SRC a truly complex injury.[36] Currently, there is no gold standard for immediate detection of SRC. With retired National Football League players reporting as many as 12 concussions in their career,[37] and as many as 50% of all National Collegiate Athletic Association (NCAA) athletes report having suffered at least 1 concussion during their university years,[38] an objective marker that could be used to identify the injury, track athlete recovery, and inform on RTP timelines would be highly welcomed by the medical community. More research is needed to identify an objective marker directly related to the pathophysiological processes that occur in the brain post-injury.

This study certainly has limitations. The sample size of the current study was small. Further, all reported SRC were mild in nature, as all athletes experienced full RTP within 3 weeks. As plasma tau and serum NF-L are elevated following SRC in individuals with PCS lasting greater than 6-days, an increase in concentration may reflect a more serious injury. No neuroimaging or biomechanical data collection took place. The 6- and 14-day time points were chosen to extend the temporal profile; however, these time points may be outside the time period to observe significant changes.[39,40]

Despite the limitations, the data presented are novel in that this is the first study to examine plasma tau and serum NF-L in the same cohort of athletes from preseason to post-concussion. Plasma tau and serum NF-L appears to remain unchanged in uncomplicated SRC but will increase in concentration in complicated SRC with persistent symptoms. More research is needed in order to identify blood-based biomarkers that can detect SRC across the entire spectrum of severity.
